# A neural network framework for similarity-based prognostics

**DOI:** 10.1016/j.mex.2019.02.015

**Published:** 2019-02-20

**Authors:** Oguz Bektas, Jeffrey A. Jones, Shankar Sankararaman, Indranil Roychoudhury, Kai Goebel

**Affiliations:** a Warwick Manufacturing Group, University of Warwick, Coventry, CV4 7AL, United Kingdom; b Pricewaterhouse Cooper, San Jose, CA, 95110, United States; c Stinger Ghaffarian Technologies, Inc., NASA Ames Research Center, Moffett Field, CA, 94035, United States; d NASA Ames Research Center, Moffett Field, CA, 94035, United States; e Luleå Technical University, Division of Operation and Maintenance Engineering, Luleå, Sweden

**Keywords:** Similarity based RUL calculation, Artificial neural networks, Data-driven prognostics

## Abstract

Prognostic performance is associated with accurately estimating remaining useful life. Difficulty in accurate prognostic applications can be tackled by processing raw sensor readings into more meaningful and comprehensive health condition indicators that will then provide performance information for remaining useful life estimations. To that end, typically, multiple tasks on data pre-processing and predictions have to be carried out such that tasks can be assessed using different methodological aspects. However, incompatible methods may result in poor performance and consequently lead to undesirable error rates.

The present research evaluates data training and prediction stages. A data-driven prognostic method based on a feed-forward neural network framework is first defined to calculate the performance of a complex system. Then, the health indicators are used in a similarity based remaining useful life estimation method. This framework presents a conceptual prognostic protocol that overcomes challenges presented by multi-regime condition monitoring data.

## Highlights

• The proposed method can provide a multi regime normalization process for noisy degradation trajectories.
• With the neural network library, it is possible to calculate health indicators of different trajectories with distinct wear levels.
• The similarity based remaining useful life estimation method can increase the prognostic performance for even short-term test subsets with little information.

## Graphical abstract

**Figure:**
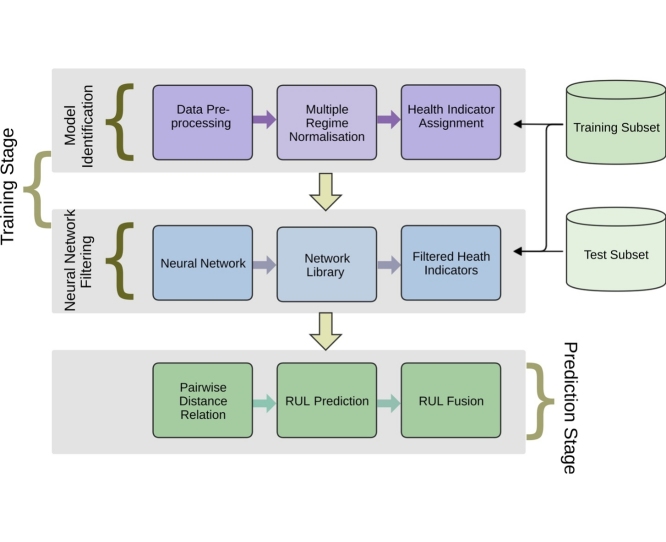

**Specifications Table**

**Table:** 

**Subject Area**	*Engineering*
**More specific subject area:**	*Prognostics and Health Management*
**Method name:**	*Feed Forward Neural Network Filtering for* *Similarity-Based Prognostics*
**Name and reference of original method**	*Oguz Bektas, Jeffrey A. Jones, Shankar Sankararaman, Indranil Roychoudhury, Kai Goebel. A Neural Network Filtering Approach for Similarity-Based Remaining Useful Life Estimations: The International Journal of Advanced Manufacturing Technology, 2018.* [1]
**Resource availability**	*The method is evaluated by PHM08 challenge data from the competition held at the 1st international conference on Prognostics and Health Management (PHM08) (Goebel, 2008). The dataset is publicly available at Data Repository of NASA Prognostics Center of Excellence.* https://ti.arc.nasa.gov/tech/dash/groups/pcoe/prognostic-data-repository/

## Method details

The complexity in accurately estimating remaining useful life (RUL) has led to a rise in the number of algorithms and methods in the prognostic literature. The significance of these methodologies relies on their potential to estimate the evolution of degradation conditions in time. Prognostics provide sufficient time for condition-based maintenance operations and notification for necessary actions. Many prognostic applications proposed for various domains can be found in the literature, such as composite materials [2], aircraft actuators [3], turbofan engines [[4], [5], [6], [7]], electronic components [8], etc.

**Fig. 1:**
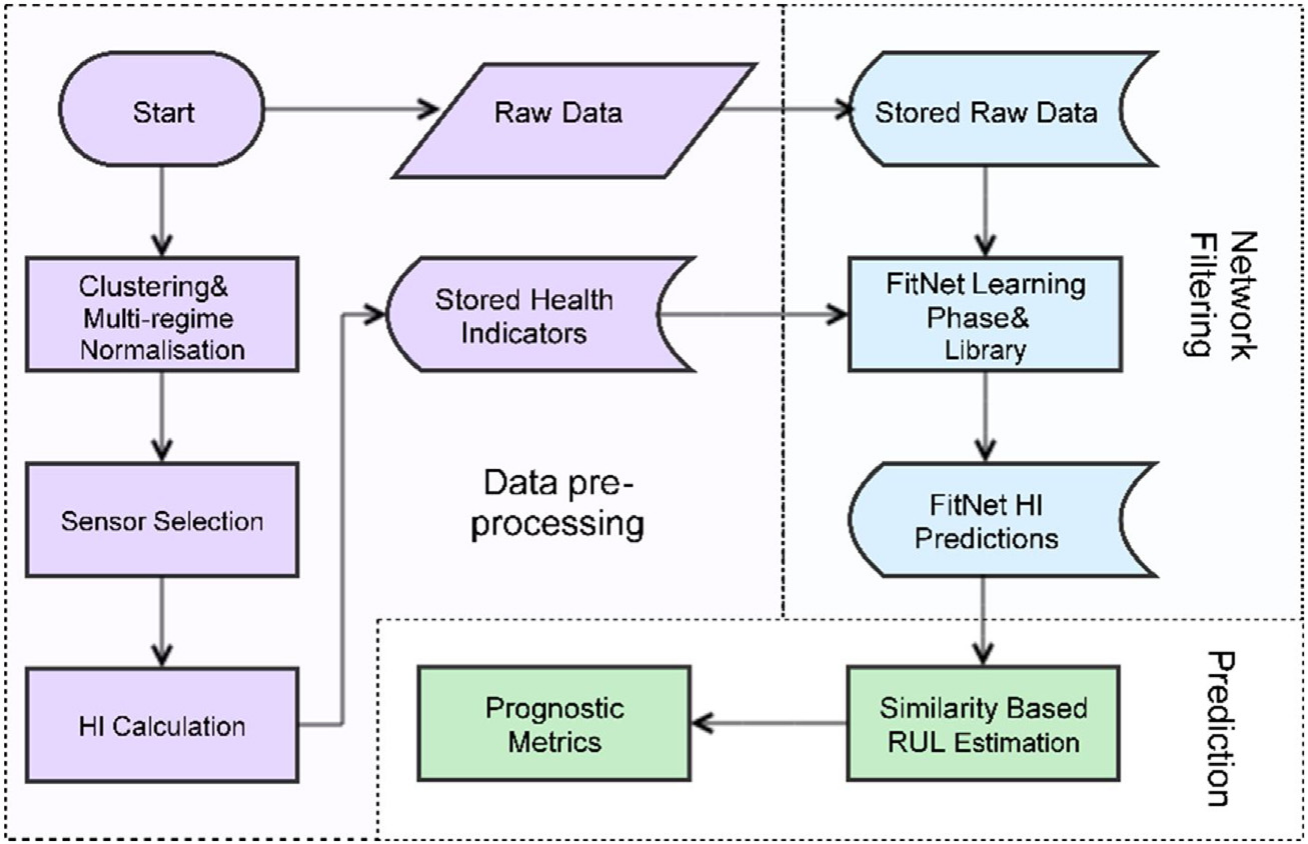
Flowchart representing the methodology stages.

In this article, the motivation is to propose a hybrid framework of data processing and RUL estimation by using various complex system indicators and multi-dimensional sensor measurements. The main research focus is based on the use of data pre-processing methods in order to increase the prognostic performance. Fig. 1 demonstrates the main stages of the protocols which has introduced a conceptual framework to overcome the challenges of multi-regime degradation behavior.

The science of prognosis is predicated on system ageing and monotonic damage accumulation, and it is possible to correlate sensor behavior with signs of ageing to estimate the remaining useful life of systems [9]. However, the multi-dimensional characteristics in raw data does not provide useful information to measure the monotonic damage accumulation. Further data processing is needed to provide useful information for remaining useful life estimations.

The first step of multi-dimensional data processing is to identify the operational regimes that can be found by finding the number of clusters in the operational settings.

(1) $$f\left(x_{p}\right)=\text{a}\text{r}{\text{g}}_{k=1,...,R}\text{f}\text{i}\text{n}\text{d}(x=k)$$

where $\text{a}\text{r}{\text{g}}_{k=1,....R}\text{f}\text{i}\text{n}\text{d}(x=k)$ defines the index of the occurrence of regimes, k, in the data *x*.

**Fig. 2:**
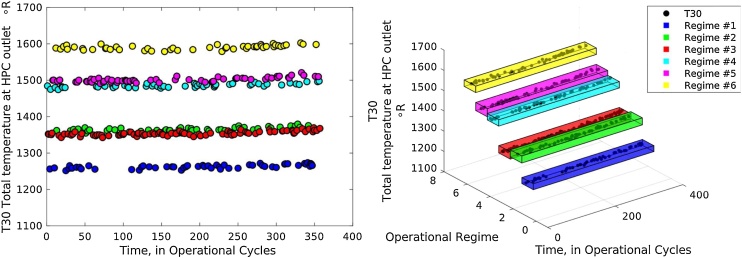
Clustered Regimes for Multi-Regime Time Series.

Fig. 2 provides an illustrative example of the multi regime sensors after regime assignment. In this sample, a multiple regime normalization method can carry out adjustments by returning raw condition monitoring measurements into a common scale (for example the z-score). To compute a z-score for each regime, it is required to know the mean (μ) and standard deviation (σ) of the regime population of each sensor to which a data point belongs.

(2) $$z=\frac{x-\mu }{\sigma }$$

This multiple regime normalization process is applied to all regimes separately; the standardized sensors are reassembled to produce a single-regime condition monitoring dataset. To produce a single health output from these normalized sensors, the multiple readings are aggregated by taking the mean of all sensor measurements at each time step.

(3) $$s_{i}=\frac{1}{n}\sum_{j=1}^{n}z_{ji}$$

where *s* is the single health output and *n* is the number of sensors.

**Fig. 3:**
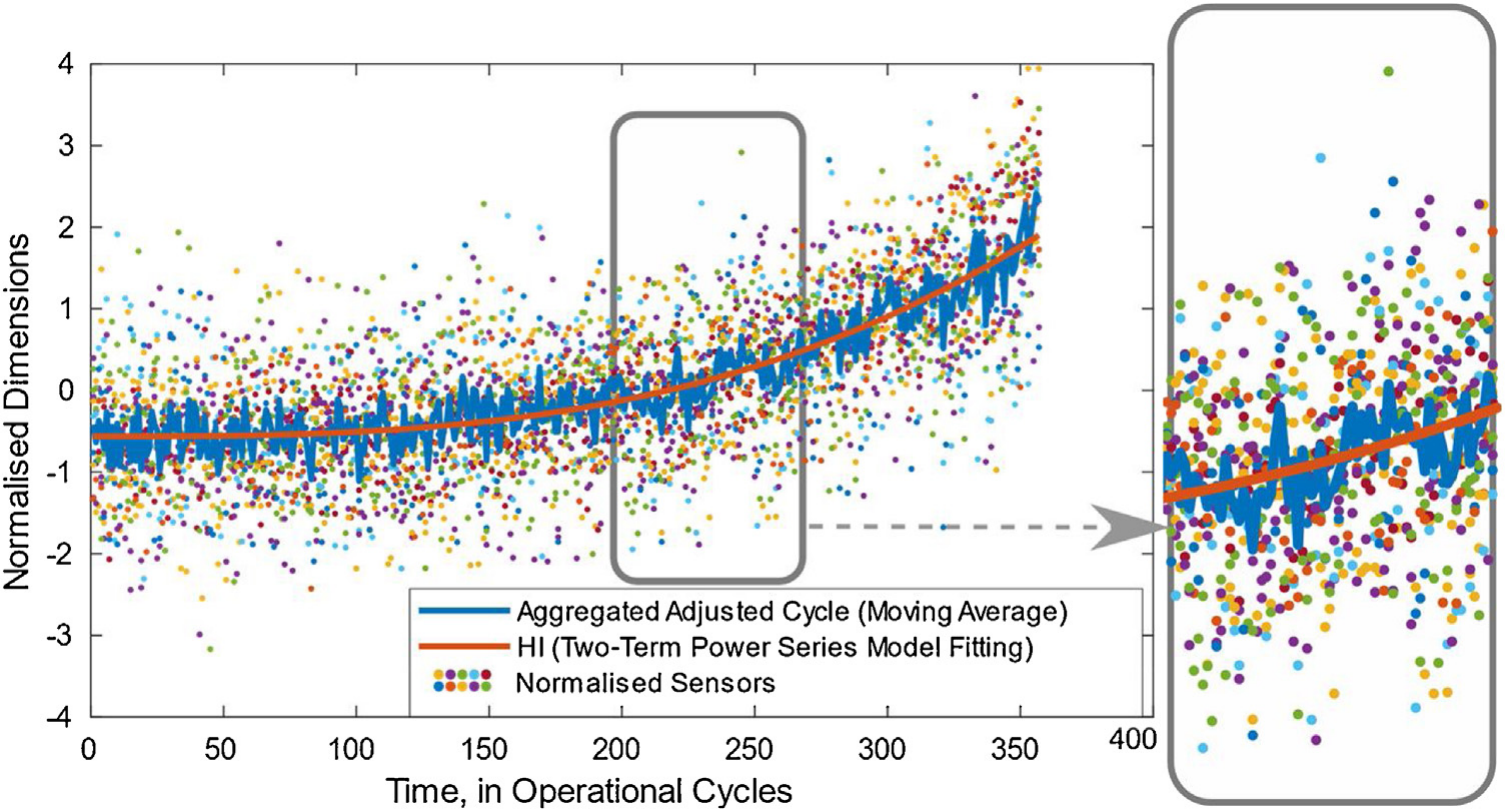
Adjusted Cycle and HI.

Even though these aggregated outputs are smoother than the normalized sensors, they are still noisy and there is a risk that when they are included in the training stage, the network might learn from the noise. Therefore, a two-term power series fitting model is applied to describe the relationship between the health output and a Health Indicator (HI) to be used in the neural network training (see Fig. 3 Adjusted Cycle and HI).

(4) $$y=as^{b}+c+\varepsilon$$

where an approximation to a power-law distribution $s^{b}$ includes two fitting terms a and *c*. The fitted HIs calculated by this fitting model exhibits only increasing values with minimal wear increase levels in early stages.

Artificial Neural Network (ANN) training is intended to accommodate the uncertainty of measurements. The parameters from the multiple regimes are handled by a network mapping process between the raw sensor values and the assigned output HI at the model identification stage. This allows the current and upcoming data to be normalized individually. The function fitting neural network is a two-layer feedforward method including a sigmoid transfer function ($f_{h}$) in the hidden layer and a linear transfer function in the output layer ($f_{0}$). This adaptive filtering model is critical for taking into account the standardization issues as well as the consistency in damage information. The general equation representing this network model is denoted by:

(5) $$y\left(t\right)=f\left[x_{\left(t\right)}\right]=f_{0}\left\{b+\sum_{h=1}^{nh}w_{h}f_{h}\left(b_{h}+\sum_{i=1}^{n}w_{ih}x_{\left(t-i\right)}\right)\right\}$$

where the state variables (*x*) are multiplied by fixed real-valued weights (*w*) and a bias (*b*) is added. The neuron's activation is obtained as a result of summation of the nodes and the nonlinear activation function (f) is applied to this sum [10,11].

**Fig. 4:**
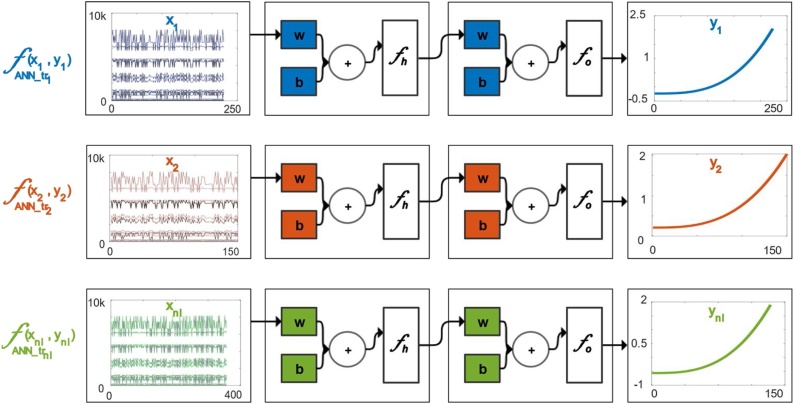
Feed Forward Neural Network Training Design.

**Fig. 5:**
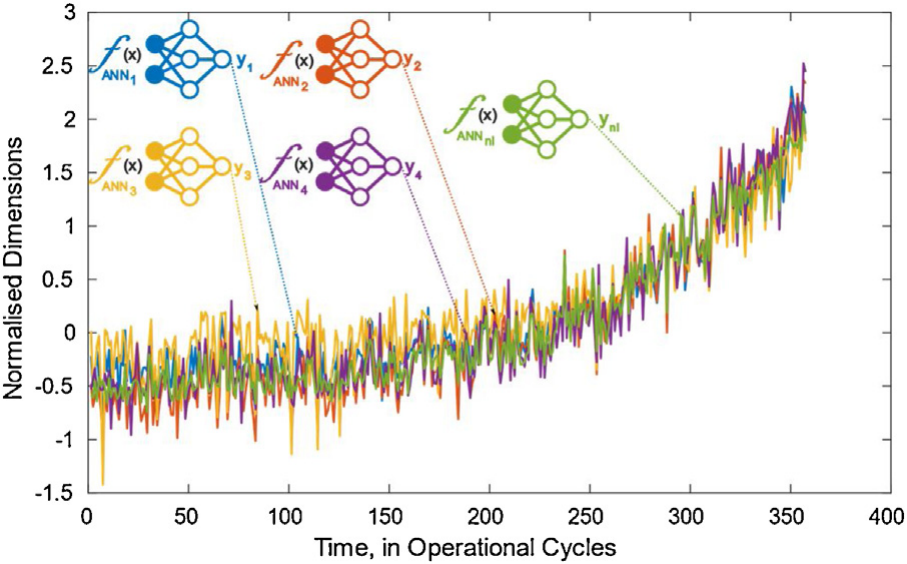
Neural Network Library Results.

Fig. 4 shows such feed-forward network training functions which take a set of input vectors (raw multidimensional condition monitoring data), and then another set of target vector (HI). Neurons, which are connected with coefficients (weights), constitute the neural network structure and evaluate the input state variables. Where a network function is trained with a single trajectory input and output, an alternative network that is trained with other input and output values, might result in different estimations due to different weight and bias coefficients. To that end, as seen on Fig. 4, multiple neural networks with their inputs and outputs from different trajectories are trained to filter the raw data and each trained function is stored in a network library with “nl” number of trained functions. This library receives all novel trajectories and produces multiple estimates for each inserted input trajectory. Fig. 5 shows a set of network library estimations that result in similar exponential growth patterns. Each line here is an estimated output of the same raw training input resulting from a certain trained network function in the library. There is a consistency between the results from different functions and similar HI estimations starting and ending at parallel wear levels are assigned adequately.

As there are multiple network estimations for a single input *“x*”), a final HI equation adapted from the moving average method is derived as follows:

(6) $$s_{i}=\left\{\begin{array}{cc} \frac{1}{p+i}\sum_{q=1}^{p+i}\frac{1}{n}\sum_{j=1}^{n}y_{jq}^{NN} & ifi-p<0\\ & \;\\ \frac{1}{p+(l-i+1)}\sum_{q=i}^{p+(l-i+1)}\frac{1}{n}\sum_{j=1}^{n}y_{jq}^{NN} & ifi+p>l\\ & \;\\ \frac{1}{2p+1}\sum_{q=r-p}^{r+p}\frac{1}{n}\sum_{j=1}^{n}y_{jq}^{NN} & ifi-p\geq 0andifi+p\leq l \end{array}\right)$$

where the final HI (s) is the mean of (p) moving average of (n) number of estimations and (l) is the length of trajectories. The window size of moving average is a numeric duration including the parameters in the current location plus previous and upcoming neighbors.

After both stages of training are achieved, the estimated health indicators are stored to calculate the pairwise distance relations for the remaining useful life predictions. A similarity-based remaining useful life estimation model is used to identify the best matching training HI units for each test HI unit. The similarity-based estimation algorithm used in this article predicts the future behavior of the systems only when there is sufficient training data to map out the damage space. HI derived from the network estimation must provide a realistic representation of system performance.

RUL estimation is applied as a tool to match the test cases with the full operational training periods. The pairwise distance between the two cases is given as a similarity measure which can be expressed as the difference between two vectors.

**Fig. 6:**
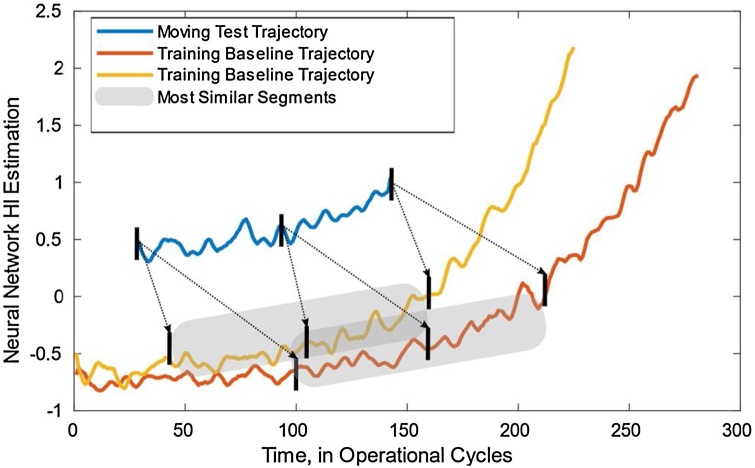
Moving Pairwise Distance Calculation.

Fig. 6 shows the moving pairwise distance calculation between a test trajectory and two full sets of training observations. The similarity for each training baseline is initially calculated at time step “1” and then, the testing curve is moved step by step to the end of the training curve to find the best-matching location that can be in the later part of the curve. Thereby, the moving pairwise distance is calculated throughout the training baseline by the following equation;

(7) $$d{\left(te,tr\right)}_{\left(j\right)}=\sum_{j=n_{te}}^{n_{tr}}\sqrt{\sum_{i=1}^{n_{te}}{\left(te_{i}-tr_{i+j}\right)}^{2}}$$

where $n_{tr}$ and $n_{te}$ respectively corresponds to the length of the training and test trajectories. Once the test cases are moved, the location of the best similar segments is identified by the minimum pairwise distance value.

(8) $$L_{te,tr}=\text{arg}\;find\left(d_{{\left(te,tr\right)}_{j}}=\text{m}\text{i}\text{n}(d_{{\left(te,tr\right)}_{\left(1\right)}},d_{{\left(te,tr\right)}_{\left(2\right)}},...,d_{{\left(te,tr\right)}_{\left(n_{tr}-n_{te}\right)}}\right);j=1,2,...,(n_{tr}-n_{te})$$

Then, these locations are used for the calculation of RUL for each baseline (training trajectory).

(9) $${RUL}_{te,tr}=n_{tr}-{(n}_{te}+L_{te,tr})$$

The final RUL is found by averaging multiple minimum distance values of baselines so that the multiple estimates could decrease the risk of biased multistep ahead estimations.

## Method validation

The algorithm is tested on PHM08 data challenge provided by the Prognostics Center of Excellence at NASA Ames, and it achieved the current overall leading score in the literature [1,12]. While the methods are designed through the application of different protocols for multi-step-ahead predictions, error based prognostic metrics are dynamically used to measure the performance of the model. The prognostic metrics, and their impact on the technical prognostic requirements, gain particular importance in the way that they complement each other. Therefore, the presented methods will be based on both the modelling and performance evaluation of prognostics in terms of technical metrics.

Before the results of the presented algorithm are sent for validation, the model is tested by reconstructed secondary test datasets which were randomly selected from full size training trajectories [13]. The length of units in reconstructed database is equal to the final test units from the original file to ensure that similar behavior of test trajectories is obtained.
